# Supplementary material for: Cover crop mixture expression is influenced by nitrogen availability and growing degree days
Source: PLoS One. 2020 Jul 27;15(7):e0235868. doi: 10.1371/journal.pone.0235868 (PMC7384630; doi:10.1371/journal.pone.0235868)

**S1 Appendix**

The custom mixes for each of the on-farm sites were designed using a combination of farmer consultation and cover crop growth data.  We first asked farmers what top 4 services they wanted from their cover crop mixtures.  Most reported N supply as the most desired service, while 4 reported weed management as the first or second desired service (**Table A**). We also asked each farmer the approximate date they anticipated planting their cover crop.

Next, we used a compiled dataset of individual cover crop species biomasses, fall precipitation, fall GDDs, initial soil %N, and seeding rates from three previous experiments conducted at Rock Springs research center and 3 nearby organic farms (Schipanski et al. 2017, Murrell et al. 2017, White et al. 2017). We used data for cereal rye to predict triticale, as cereal rye is a parent species of triticale and we had more data on cereal rye performance in mixtures than triticale data.  We ran multiple regressions to determine the relationships between the environmental factors and biomass production of each individual cover crop species (**Table B**). We used a square root transformation on biomass of each cover crop species so the data fit the assumptions of the regression models.

The significant model parameters from each of the regressions were then used to design a biomass predictor tool (**Fig. A**). Historical cumulative GDD data from NOAA by farm location, along with the farmer’s planting date information and soil organic matter (which we had for their fields) were used to create fixed environmental condition parameters (precipitation, GDD, soil %N) for each farm in the biomass tool.  We then manipulated the seeding rates of each of the cover crop mixture species in the biomass tool to predict how the fall species composition and biomasses would look for that mixture at the specified farm (**Fig. A**).

No seeding rate was set lower than 5% of monoculture seeding rate for any of our cover crop species.  At the same time, the biomass tool predicted high fall canola biomass in the standard mix at most of the farms. Therefore, most “farm-tuned” mixtures had the minimum canola seeding rate of 5% monoculture rate.  Seeding rates of the mixtures were then further adjusted to either favor more legume biomass if nitrogen supply was the top priority of the farmer, or more legume and triticale biomass if the farmer had indicated weed management as a high priority.

**References**

Finney DM, EG Murrell, CM White, B Baraibar, ME Barbercheck, BA Bradley, S Cornelisse,

MC Hunter, JP Kaye, DA Mortensen, CA Mullen, ME Schipanski. 2017. Ecosystem

services and disservices are bundled in simple and diverse cover cropping systems.

*Agricultural & Environmental Letters* 2:170033. DOI: 10.2134/ael2017.09.0033

Murrell EG, ME Schipanski, DM Finney, MC Hunter, M Burgess, JC LaChance, B Baraibar,

CM White, DA Mortensen, JP Kaye. 2017. Achieving diverse cover crop mixtures:

Effects of planting date and seeding rate. *Agronomy Journal* 109:259-271.

White CM, ST DuPont, M Hautau, D Hartman, DM Finney, B Bradley, JC LaChance, JP Kaye.

2017. Managing the trade off between nitrogen supply and retention with cover crop

mixtures. *Agriculture, Ecosystems, & Environment* 237:121-133.

**Table A. Ranking of services desired for the custom cover crop mixture at each on-farm site, as specified by the farmer.**

|  | **1^st^ Service** | **2^nd^ Service** | **3^rd^ Service** | **4^th^ Service** |
| --- | --- | --- | --- | --- |
| Farm 1 | Weed Management | Reduce N Leaching | N Supply | Build OM |
| Farm 2 | N Supply | Weed Management | Build OM | Beneficial Insects |
| Farm 3 | N Supply | Reduce N Leaching | Build OM | Weed Management |
| Farm 4 | N Supply | Build OM | Weed Management | Beneficial Insects |
| Farm 5 | N Supply | Build OM | Reduce N Leaching | Weed Management |
| Farm 6 | Weed Management | N Supply | Build OM | Reduce N Leaching |
| Farm 7 | N Supply | Weed Management | Build OM | Forage for Cattle |
| Farm 8 | N Supply | Build OM | Weed Management | Reduce N Leaching |

**Table B.** **Multiple regression results for fall biomass of our 5 cover crop species as predicted by fall GDDs, initial soil % N, and seeding rate.**

| **Model Parameter** | **Canola** | | | **Cereal Rye**  **(Triticale)** | | | **Crimson clover** | | | **Red clover** | | | **Austrian**  **Winter Pea** | | |
| --- | --- | --- | --- | --- | --- | --- | --- | --- | --- | --- | --- | --- | --- | --- | --- |
|  | **df** | **F** | **p** | **df** | **F** | **p** | **df** | **F** | **p** | **df** | **F** | **p** | **df** | **F** | **p** |
| Seeding Rate | 1,56 | 10.81 | 0.0017 | 1,76 | 74.64 | <0.0001 | 1,64 | 16.63 | 0.0001 | 1,32 | 48.86 | <0.0001 | 1,83 | 83.52 | <0.0001 |
| Soil %N | 1,56 | 29.73 | <0.0001 | 1,76 | 8.08 | 0.0058 | 1,64 | 11.61 | 0.0011 | 1,32 | 8.69 | 0.0059 | 1,83 | 75.71 | <0.0001 |
| Fall GDD | 1,56 | 2.35 | 0.1308 | 1,76 | 13.32 | 0.0005 | 1,64 | 34.68 | <0.0001 | 1,32 | 11.84 | 0.0016 | 1,83 | 11.14 | 0.0013 |
|  |  |  |  |  |  |  |  |  |  |  |  |  |  |  |  |
| **R^2^** | 0.4133 | | | 0.5945 | | | 0.5260 | | | 0.8010 | | | 0.6416 | | |

**Figure A. Biomass predictor tool.** The biomass predictor tool used to predict the fall biomass amount and relative composition of the standard mixture (left pie chart), as well as determine the seeding rates needed to create a custom (“farm-tuned”) mixture with more even distribution of biomass by cover crop species (right pie chart).


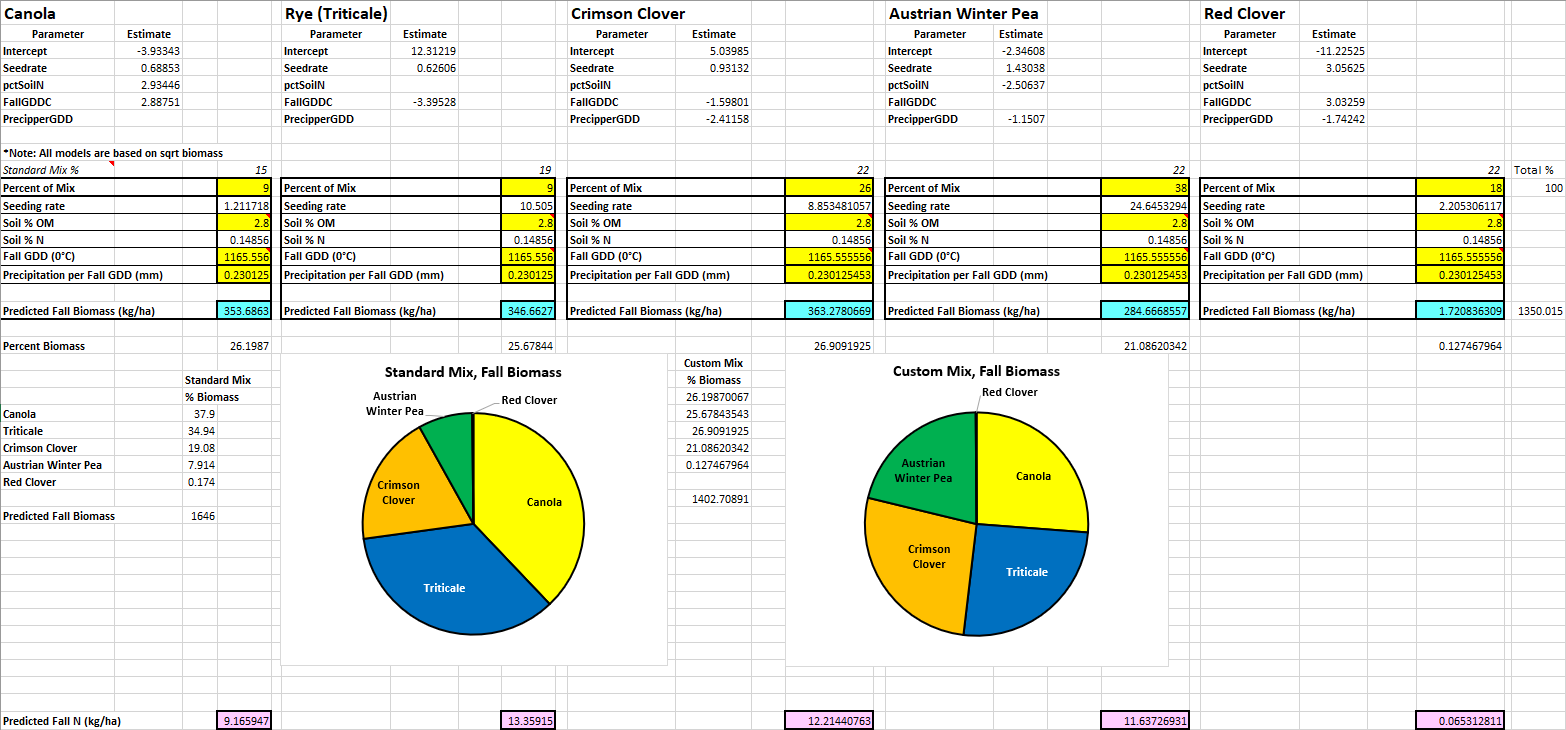

Supplement: S1 Appendix — (DOCX) [file pone.0235868.s001.docx]
