# Supplementary material for: Cover crop mixture expression is influenced by nitrogen availability and growing degree days
Source: PLoS One. 2020 Jul 27;15(7):e0235868. doi: 10.1371/journal.pone.0235868 (PMC7384630; doi:10.1371/journal.pone.0235868)
Supplement: S2 Appendix — (DOCX) [file pone.0235868.s002.docx]

**Appendix 2**

**Table A**. **On-farm cover crop mixture seeding dates and climatic and soil conditions**

|  |  | 2016 - 2017 | | | | | 2017-2018 | | | | |
| --- | --- | --- | --- | --- | --- | --- | --- | --- | --- | --- | --- |
| Farm location | Farm-tuning | Seeding date 2016 | Term. date 2017 | Fall GDD | Spring GDD | Soil iN ** | Seeding date 2017 | Term. date 2018 | Fall GDD | Spring GDD | Soil iN** |
| Farm 1 | L+T+ | 8/3 | 5/1 | 785 | 427 | 4.3 | 8/22 | 5/8 | 1301 | 604 | 20.1 |
| Farm 2 | L+T+ | 8/1 | 5/18 | 1520 | 887 | 6.5 | 8/1 | 5/18 | 1552 | 610 | 6.8 |
| Farm 3 | L+T+ | 8/4 | 4/17 | 1713 | 473 | 14.2 | 8/29 | 5/14 | 1112 | 608 | 3.7 |
| Farm 4 | L+T+ | 8/19 | 4/24 | 1274 | 566 | 21.9 | 8/25 | 5/2 | 1045 | 409 | 10.5 |
| Farm 5 | L+T- | 9/1 | 5/11 | 755 | 533 | 45.2 | 8/22 | 5/9 | 945 | 342 | 69.7 |
| Farm 6 | L+T- | 9/8 | 4/27 | 1038 | 791 | 29.2 | 10/2 | 4/30 | 620 | 573 | 5.9 |
| Farm 7 | L+T- | 8/16 | 5/16 | 1306 | 789 | 27.6 | 8/11 | 5/21 | 1324 | 641 | 15.7 |
| Farm 8 | L+T- | 9/6 | 4/11 | 1056 | 559 | 12.6 | 9/5 | 4/20 | 1165 | 455 | 5.0 |

** averaged over 4 blocks

Cover crop mixture seeding and termination dates, fall GDD (from planting to fall biomass sampling), spring GDD (from 1 January to termination) and soil iN (NO_3_^-^ -N plus NH_4_^+^ -N in units of mg N kg^-1^ dry soil) at cover crop planting . L+T+ farms increased legume and triticale seeding rates, L+T- farms increased legume but decreased triticale seeding rates. All farms decreased canola seeding rates.

**Table B**. **Farm site soil type descriptions**

| **Farm location** | **Soil Type**  **(Taxonomic class -USDA soil survey staff)** | **Min temperature (°C)** | **Max temperature (°C)** | **Precipitation (August – May, cm)** | **Approx.Latitude** | **Approx. Longitude** | **County, State** |
| --- | --- | --- | --- | --- | --- | --- | --- |
| **Farm 1**  Year 1  (2016-2017) | Shaker loam (coarse-loamy over clayey, mixed, semiactive, nonacid, mesic Aeric Epiaquepts) underlies approximately 85 % of the study site, with the remainder consisting of Collamer silt loam (fine-silty, mixed, semiactive, mesic Glossaquic Hapludalfs). | -21 | 33 | 103 | 42.1 | -73.8 | Columbia Co., NY. |
| Year 2  (2017-2018) | Knickerbocker fine sandy loam soil (sandy, mixed, mesic Typic Dystrudepts) underlies approximately 97.9% of the study site, with the remainder consisting of Manlius channery silt loam soil (loamy-skeletal, mixed, active, mesic Typic Dystrudepts). | -22 | 31 | 65 | 42.2 | -73.8 |  |
| **Farm 2**  Year 1 | Gladstone gravelly loam ( fine-loamy, mixed, active, mesic Typic Hapludults) underlies approximately 85% of the study site, with the remainder consisting of Parker gravelly loam (loamy-skeletal, mixed, semiactive, mesic Typic Dystrudepts) | -13 | 35 | 82 | 40.0 | -75.8 | Chester Co., PA |
| Year 2 | Gladstone gravelly loam soil (fine-loamy, mixed, active, mesic Typic Hapludults) underlies approximately 80% of the study site, with the remainder consisting of Califon loam (fine-loamy, mixed, active, mesic Typic Fragiudults - 15%), and Parker gravelly loam  (loamy-skeletal, mixed, semiactive, mesic Typic Dystrudepts . | -18 | 34 | 93 |  |  |  |
| **Farm 3**  Year 1 | Hagerstown silt loam (fine, mixed, semiactive, mesic Typic Hapludalfs)  underlies approximately 50% of the study site, with the remainder consisting of Duffield silt loam (fine-loamy, mixed, active, mesic Ultic Hapludalfs - 30%) and Hagerstown silty clay loam (20%) | -14 | 34 | 74 | 40.2 | -76.4 | Lancaster Co. PA |
| Year 2 | Same as Yr 1. | -19 | 32 | 89 |  |  |  |
| **Farm 4**  Year 1 | Hazleton channery loam (loamy-skeletal, siliceous, active, mesic Typic Dystrudepts) underlies approximately 90% of the study site, with the remainder consisting of Cavode silt loam (fine, mixed, active, mesic Aeric Endoaquults) | -17 | 33 | 110 | 40.9 | -80.0 | Butler Co. PA |
| Year 2 | Same as Yr 1. | -20 | 32 | 100 |  |  |  |
| **Farm 5**  Year 1 | Lansing gravelly silt loam (fine-loamy, mixed, active, mesic Glossic Hapludalfs) underlies approximately 50% of the study site, with the remainder consisting of Conesus gravelly silt loam (fine-loamy, mixed, active, mesic Glossaquic Hapludalfs 28%), Kendaia (fine-loamy, mixed, semiactive, nonacid, mesic Aeric Endoaquepts) and Lyons (fine-loamy, mixed, active, nonacid, mesic Mollic Endoaquepts) soils (22%). | -21 | 33 | 88 | 42.5 | -76.7 | Tompkins Co., NY |
| Year 2 | Howard gravelly loam soil (loamy-skeletal, mixed, active, mesic Glossic Hapludalfs) underlies majority (99%) of the study site. | -23 | 32 | 73 | 42.4 | -76.6 |  |
| **Farm 6**  Year 1 | Ernest silt loam (fine-loamy, mixed, superactive, mesic Aquic Fragiudults) underlies approximately 53% of the study site, with the remainder consisting of Brinkerton silt loam (fine-silty, mixed, superactive, mesic Typic Fragiaqualfs - 47%). | -12 | 36 | 75 | 40.6 | -77.4 | Juniata Co., PA |
| Year 2 | Tyler silt loam soil (fine-silty, mixed, active, mesic Aeric Fragiaquults) underlies approximately 45% of the study site, with the remainder consisting of Purdy silt loam (fine, mixed, active, mesic Typic Endoaquults - 25%), and Monongahela silt loam (fine-loamy, mixed, semiactive, mesic Typic Fragiudults - 30%). | -18 | 33 | 94 |  |  |  |
| **Farm 7**  Year 1 | Berks-Weikert complex (Berks - loamy-skeletal, mixed, active, mesic Typic Dystrudepts, Weikert - loamy-skeletal, mixed, active, mesic Lithic Dystrudepts) underlies approximately 70% of the study site, with the remainder consisting of Comly silt loam (fine-loamy, mixed, active, mesic Oxyaquic Fragiudalfs). | -14 | 33 | 85 | 40.7 | -75.6 | Lehigh Co., PA |
| Year 2 | Berks-Weikert complex underlies approximately 100% of the study site | -22 | 30 | 110 |  |  |  |
| **Farm 8**  Year 1 | Edom complex (fine, illitic, mesic Typic Hapludalfs) underlies approximately 90% of the study site, with the remainder consisting of Washington silt loam (fine-loamy, mixed, semiactive, mesic Ultic Hapludalfs). | -15 | 33 | 81 | 41.0 | -76.8 | Montour Co., PA |
| Year 2 | Same as Year 1 | -20 | 32 | 77 |  |  |  |
| Research Station  Year 1 | The dominant soil type at this location is a Hagerstown silt loam (Taxonomic class: fine, mixed, semiactive, mesic Typic Hapludalf0 | -16 | 32 | 88 | 40.7 | -77.9 | Centre Co., PA |
| Year 2 | Same as Year 1 | -23 | 30 | 89 |  |  |  |

Source for soil type: Taxonomic class-USDA survey staff, minimum and maximum growing season temperatures (August to May, °C), accumulated precipitation (August to May, cm), approximate farm locations, county and state. Separate for year 1 (2016 to 20017), and year 2 (2017 to 2018).

**References**

Soil Survey Staff. 1999. Soil taxonomy: A basic system of soil classification for making and interpreting soil surveys. 2nd edition. Natural Resources Conservation Service. U.S. Department of Agriculture Handbook 436.

NOAA Northeast Regional Climate Centers. Northeast RCC climrod2, NOAA regional climate centers ACIS. 10.5.2020 <http://climod2.nrcc.cornell.edu/>. accessed 1/06/2020.
